# Association of Attention Deficit/Hyperactivity Disorder With Events Occurring During Pregnancy and Perinatal Period

**DOI:** 10.3389/fpsyg.2021.707500

**Published:** 2021-09-21

**Authors:** Jianbo Liu, Yuqiong He, Yanmei Shen, Yuanyue Zhou, Tiantian Meng, Bo Xiao, Xilong Cui, Yumin Fang, Jianping Lu, Yu-Tao Xiang, Xuerong Luo

**Affiliations:** ^1^Department of Child Psychiatry of Shenzhen Kangning Hospital, Shenzhen Mental Health Center, School of Mental Health, Shenzhen University, Shenzhen, China; ^2^National Clinical Research Center for Mental Disorders, Department of Psychiatry, The Second Xiangya Hospital of Central South University, Changsha, China; ^3^Hunan Key Laboratory of Psychiatry and Mental Health, Changsha, China; ^4^Mental Health Zhejiang University School of Medicine, Hangzhou Seventh People’s Hospital, Hangzhou, China; ^5^Unit of Psychiatry, Department of Public Health and Medicinal Administration, Institute of Translational Medicine, Faculty of Health Sciences, University of Macau, Macao, SAR China

**Keywords:** ADHD, threatened abortion, low birth weight, neonatal asphyxia, instrumental delivery

## Abstract

**Background:** The relationship of events occurring during pregnancy and perinatal period with attention deficit/hyperactivity disorder (ADHD) is not clear. Thus, the focus of the current study was to examine the effects of events occurring during pregnancy and perinatal period on ADHD.

**Methods:** A two-phase cross-sectional study was performed across 13 schools in Changsha and Yiyang cities from March to December, 2014. We preliminarily screened all students using CBCL and established the diagnosis using Mini International Neuropsychiatric Interview for Children and Adolescents (MINI-KID) and the Diagnostic and Statistical Manual of Mental Disorders (DSM-IV). A total of 3,418 questionnaires were effectively completed in this study.

**Results:** History of threatened abortion (TA) [odds ratio (OR): 1.707 (1.201–2.426)] (vs. No-TA) and neonatal asphyxia (NA) [OR: 2.497(1.225–5.09)] (vs. health) showed a positive association with ADHD. On subgroup analysis, TA [OR: 2.216 (1.458–3.369)] (vs. No-TA) was a risk factor for ADHD without comorbidity; instrumental delivery [OR: 2.748 (1.057–7.142)] (vs. natural birth) and NA [OR: 2.789 (1.222–6.361)] (vs. health) were risk factors for ADHD in the subgroup of ADHD with comorbidity; TA (vs. no-TA) and NA (vs. health) were risk factors for ADHD among male students [ORs: 2.232 (1.439–3.462) and 2.808 (1.115–7.068), respectively], while low birth weight (LBW) (vs. normal birth weight) was a risk factor [OR: 2.054 (1.063–3.967)] for ADHD among female students.

**Conclusion:** TA was a risk factor for ADHD in the absence of comorbid conditions; instrumental delivery and NA were risk factors for ADHD in the subgroup of ADHD with comorbidity; TA and NA were risk factors for ADHD among male students. LBW was a risk factor for ADHD among female students.

## Background

Attention deficit/hyperactivity disorder (ADHD) is a common neurodevelopmental disorder characterized by hyperactivity, impulsive behavior, and poor attention (Choi et al., 2017). According to a meta-analysis, an estimated 5.9–7.1% of children and adolescents are affected by ADHD globally; the estimates were based on ratings by parents/teachers or on clinical diagnosis (Willcutt, 2012). In our previous study, the estimated prevalence of ADHD among children and adolescent students in Hunan province, China was found to be 4.64–5.29%, according to the Diagnostic and Statistical Manual of Mental Disorders (DSM-IV) (Shen et al., 2018a). Based on the assumption that ADHD may persist to adulthood in a certain proportion of individuals, we also investigated the prevalence of ADHD among Chinese medical college students and found similar prevalence rate (3.02–3.98%) (Shen et al., 2018b); this indicates that ADHD is not limited to children and adolescents. Students with ADHD may have poor executive function (Capri et al., 2020; Fabio et al., 2020), experience academic difficulties (Martin, 2014) and are at a higher risk of injuries (Amiri et al., 2017). In addition, individuals with ADHD often have comorbid psychiatric disorders, such as anxiety disorder, disruptive behavioral disorders, oppositional defiant disorder, sleep disorder, and substance use disorder (Bilgiç et al., 2013; Miguel et al., 2016; Reale et al., 2017). Generally, individuals with ADHD who have comorbid psychiatric disorders have poorer mental health. For example, Miguel et al. (2016) found that subjects with ADHD who had concomitant substance use disorder had poorer cognitive function as compared with ADHD without substance use disorder. In a study, adult subjects with ADHD who had concomitant substance use disorder showed lower gray matter volume in striatum as compared to their counterparts with only ADHD, which may be an underlying pathogenic mechanism (van Wingen et al., 2013). Therefore, ADHD is a major global public health problem that imposes a considerable socioeconomic burden. Doshi et al. (2012) found that the overall national annual incremental costs attributable to ADHD in the United States were in the range of $143–$266 billion.

However, the pathogenic mechanism of ADHD is not clear. Previous studies have found linkage of ADHD with many factors including genetic and environmental factors. In previous studies, some genes related to the dopamine pathway were shown to be associated with ADHD (Nyman et al., 2007). With respect to environmental factors, change in household income, home environment, and parental rearing style were shown to be associated with ADHD (Tully et al., 2004; Rodriguez and Bohlin, 2005; Mulligan et al., 2013; Arijit et al., 2016; Dadds et al., 2016). In addition, the role of certain gestation-related risk factors in the pathogenesis of ADHD has attracted attention. For example, maternal stress, anxiety, depression, sleep disorder, infection, lower maternal levels of vitamin D, and smoking during pregnancy showed a significant association with ADHD (Rodriguez and Bohlin, 2005; Ronald et al., 2010; Zhu et al., 2014; Morales et al., 2015; He et al., 2017; Laugesen et al., 2017; Ginsberg et al., 2018; Vizzini et al., 2018). Such associations pertaining to the perinatal period are also of particular concern. In addition, perinatal period is associated with a greater risk of brain injury due to causes such as intrapartum hypoxic ischaemia and brain injury during assisted modes of delivery. For example, Rennie et al. (2007) found an association between intrapartum hypoxic ischaemia and a range of childhood disabilities, including cognitive deficit. Moreover, neonatal asphyxia has also been shown to be a risk factor for psychiatric disorders such as ADHD and autism (Li et al., 2011; Duan et al., 2014). In addition, low birth weight and threatened abortion [before the fetus would be viable ex utero, vaginal bleed with a closed cervix (Greene, 2019)] may also be risk factors for psychiatric disorders (Hanć et al., 2015; Wang et al., 2017). Although the above studies found an association of ADHD with some events occurring during pregnancy and the perinatal period, some other studies have yielded inconsistent results (Oudin et al., 2019; O’Callaghan and Harvey, 1997). This may be attributable to small sample size in some studies which may limit the statistical power of the analysis. Secondly, subjects with ADHD frequently have other comorbid psychiatric disorders, which may have affected the results of some of the studies.

Based on the limitation of prior studies, we hypothesized that certain pregnant and perinatal events were independent risk factors for ADHD. To test this hypothesis, we assessed the relationship between ADHD and events occurring during pregnancy and perinatal period in a large sample after controlling for other possible confounding factors such as parental rearing style and demographic characteristics. In addition, we hypothesized that risk factors for ADHD pertaining to pregnancy and the perinatal period may vary depending on the sex as well as the clinical profile of ADHD (e.g., ADHD alone versus ADHD with other comorbid mental disorders). To test this hypothesis, we performed subgroup analysis in subgroups of ADHD disaggregated by comorbidity and sex to further explore the risk factors for ADHD.

## Materials and Methods

### Design and Population

This study used data from the epidemiological investigation of Hunan children and adolescent psychiatry disorders (age range of subjects: 6–16 years) from March to December, 2014 (Shen et al., 2018a). This study is a two-stage study, including screening of children with Child Behavior Checklist (CBCL) and establishment of diagnosis using the Mini International Neuropsychiatric Interview for Children and Adolescents (MINI-KID) and DSM-IV. We screened all students (*n* = 18,778) in 13 primary and junior high schools of Changsha and Yiyang cities using the CBCL. A total of 17,071 students qualified the selection criteria and were included in the study, while 1707 students were excluded. The reasons for exclusion were: refusal to complete the questionnaire or incomplete questionnaire (*n* = 782), questionnaire with >10% missing data (*n* = 522), and age outside the range of 6–16 years (*n* = 403). If the score of any subscale in CBCL is higher than the norm, it is regarded as CBCL positive. According to the results of CBCL, students who received a positive CBCL score (*n* = 3465) and matched students who received a negative CBCL score (*n* = 3465) were selected from the 17,071 students; these students were asked to complete the investigation of parental rearing attitude and behavior and their parents were asked to complete the investigation of events related to pregnancy and the perinatal period of their children. Finally, a total of 3,418 questionnaires were effectively completed for the investigation of events related to pregnancy and the perinatal period and the egma minen bardoms uppfostran (response rate: 49.29%) among students who received a positive CBCL score (*n* = 3465) and matched students who received a negative CBCL score (*n* = 3465). Further, students who received a positive CBCL score (*n* = 3465) and 10% of students who were CBCL negative (randomly sampled) among 17071 students were diagnosed using MINI-KID and DSM-IV; of these, 1,663 students (age range, 6–16 years) were confirmed to have a diagnosis of a psychiatric disorder. Among students (*n* = 3418) for whom the investigation of events related to pregnancy and the perinatal period and the egma minen bardoms uppfostran was effectively completed, 790 students were diagnosed with ADHD (including 383 who were diagnosed with ADHD comorbid with other mental disorders), 780 students were diagnosed with mental disorders other than ADHD, and 1,848 students were identified as healthy based on MINI-KID and DSM-IV. The Ethics Committee at the Second Xiangya Hospital approved this study. All parents or guardians and students in this study gave written informed consent.

### The Screening and Diagnosis Tool

#### Measurement of Demographic Variables and Variables Related to Pregnancy and the Perinatal Period

Demographic variables include age of students, gender of students (male/female), place of residence (city/semi-urban areas/village/migrants from countryside residing in city), single-child (yes/no), father’s education level and mother’s education level (Primary school educational level and below/Junior high school level/High school level/Junior college level/University education level/Postgraduate level). Events related to pregnancy and the perinatal period (from parent reports) included maternal health status during pregnancy (healthy/hypertension of pregnancy/other), threatened abortion (no/yes), mode of delivery (natural birth/Cesarean delivery/instrumental delivery), newborn health (healthy/neonatal asphyxia/other), birth weight (normal birth weight/low birth weight/macrosomia), and feeding patterns (breastfeeding/artificial feeding/mixed feeding).

#### The Child Behavior Checklist

In this study, the section of the child behavior checklist (CBCL) (a 113-item standardized scale) pertaining to behavioral problems was used to quantify behavioral problems (including internalization and externalization) of children in the age-group of 6–16 years. Each item in this section is scored as 0, 1 or 2. The Chinese version of the CBCL has been validated with satisfactory psychometric properties (retest reliability: 0.77–0.79; criterion validity: 0.61–0.76) (Su and Li, 1996).

#### The Mini International Neuropsychiatric Interview for Children and Adolescents 5.0 (MINI-KID)

The MINI-KID, a structured interview instrument to diagnose psychiatric disorders among subjects in the age-group of 6–16 years, was designed according to DSM-IV and the 10th revision of the International Statistical Classification of Diseases and Related Health Problems (ICD-10). Both the Chinese parent and child versions of the MINI-KID have been shown to have satisfactory validity (Liu et al., 2010, 2011). In this study, ADHD identified based on either parent version or child version was further verified clinically to confirm the diagnosis as per the DSM-IV.

#### Egma Minnen Barndoms Uppfostran

The Egma Minnen Barndoms Uppfostran (EMBU) is an 81-item self-reported scale that is widely used to assess the subject’s memories of parental rearing attitude and behavior. The items are rated on a four-point scale ranging from 1 (no, never) to 4 (yes, always) for the father and the mother separately. The EMBU consists of 11 factors including father rearing attitude and behavior (6 factors) and mother rearing attitude and behavior (5 factors). The Chinese version of EMBU used in this study has been validated in Chinese adolescents with correlation coefficient between factors and total scale scores ranging from 0.34 to 0.65; the Cronbach’s alpha ranges from 0.65 to 0.86 (Lai et al., 2013).

### Statistical Analysis

Data analysis was conducted using SPSS version 21 (IBM, Armonk, NY, United States). Continuous variables are presented as mean (*M*) ± standard deviation (SD), and categorical variables are presented as frequency (%). The association between each research variable and ADHD was tested individually using univariate analyses (logistic regression analysis) and odds ratios (OR) with 95% confidence intervals (CIs) calculated. We also performed multivariate analysis (logistic regression analysis) by including variables that qualified the following criteria: variables that showed a significant association with ADHD (*p* < 0.1) in univariate analyses; variables meeting clinical plausibility despite not showing a significant association in univariate analyses. For multivariate analysis, we employed two models. In the first model, we included events occurring during pregnancy and perinatal period in the logistic regression model. In the second model, we included demographic variables and parental rearing style on the basis of the first model. In addition, subgroup analysis was also performed by disaggregating subjects with ADHD by comorbidity and gender to explore the association of different subgroups with events occurring during pregnancy and perinatal period in the above two models. *P*-values in all analyses were two sided, with a significance level of 0.05.

## Results

### Demographic Characteristics and Univariate Analysis

The demographic characteristics are summarized in Table 1. On univariate analysis, female gender, younger age of students, maternal age 40-49 years (vs. 20-29 years), father’s emotional warmth, and mother’s emotional warmth were found to protect against ADHD compared with the reference group (*p* < 0.05 for all). Further, variables such as migrants from countryside living in cities (vs. city), father punishment, father interference, father rejection, father overprotection, mother emotional warmth, mother interference, mother rejection, mother punishment, single child (vs. non-single child), hypertension of pregnancy (vs. health), threatened abortion (TA) (vs. no-TA), cesarean delivery (vs. natural birth), instrumental delivery (vs. natural birth), neonatal asphyxia (NA) (vs. health), and low birth weight (LBW) (vs. normal birth weight) were found to be risk factors for ADHD (all *p* < 0.05) (Table 2).

**TABLE 1:** Demographic data for ADHDs and healthy controls.

Variables	ADHD (*n* = 790)		Healthy controls (*n* = 1848)	
Age of students	11.01 ± 2.69 years		12.14 ± 2.61 years	
Gender of students				
–Male	577	73.04%	906	49.03%
–Female	213	26.96%	942	50.97%
**Place of residence**				
–City	264	33.42%	605	32.74%
–Semi-urban areas	128	16.20%	345	18.67%
–Village	286	36.20%	707	38.26%
–Migrants from countryside residing in city	112	14.18%	191	10.33%
**Single-child**				
–Yes	318	40.25%	633	34.25%
–No	472	59.75%	1215	65.75%
**Father’s education level**				
–Primary school educational level and below	127	16.07%	298	16.13%
–Junior high school level	335	42.41%	742	40.15%
–High school level	202	25.57%	429	23.21%
–Junior college level	64	8.10%	174	9.42%
–University education level	51	6.46%	163	8.82%
–Postgraduate level	11	1.39%	42	2.27%
**Mother’s education level**				
–Primary school educational level and below	156	19.75%	337	18.24%
–Junior high school level	325	41.14%	786	42.53%
–High school level	196	24.81%	455	24.62%
–Junior college level	49	6.20%	121	6.55%
–University education level	49	6.20%	130	7.03%
–Postgraduate level	15	1.90%	19	1.03%

*ADHD: Attention deficit/hyperactivity disorder.*

**TABLE 2:** Results of univariate analysis (logistic regression analysis) for predicting ADHD in the total study population (*n* = 2638).

Variables		*P*	OR	95% CI	
Gender of students	Male		Reference		
	Female	**<0.001**	**0.355**	**0.296**	**0.426**
Age of students		**<0.001**	**0.854**	**0.828**	**0.882**
Father’s age	20–29		Reference		
	30–39	0.842	1.128	0.345	3.69
	40–49	0.786	0.849	0.26	2.772
	≥50	0.808	1.17	0.329	4.164
Father’s education level	Primary school educational level below		Reference		
	Junior high school level	0.644	1.059	0.83	1.353
	High school level	0.464	1.105	0.846	1.443
	Junior college level	0.415	0.863	0.606	1.23
	University education level	0.108	0.734	0.504	1.07
	Postgraduate level	0.17	0.615	0.307	1.232
Mother’s age	20–29		Reference		
	30–39	0.11	0.627	0.354	1.111
	40–49	**0.028**	**0.523**	**0.294**	**0.933**
	≥50	0.416	0.644	0.224	1.857
Mother’s education level	Primary school educational level below		Reference		
	Junior high school level	0.335	0.893	0.71	1.124
	High school level	0.577	0.931	0.722	1.199
	Junior college level	0.493	0.875	0.597	1.282
	University education level	0.288	0.814	0.557	1.19
	Postgraduate level	0.137	1.705	0.844	3.445
Family economic level	Very wealthy families		Reference		
	Relatively wealthy families	**0.05**	**0.482**	**0.232**	**1.001**
	Moderate families	0.085	0.538	0.266	1.089
	Less well-off families	0.575	0.800	0.366	1.746
	Very poor families	0.107	3.429	0.765	15.358
Place of residence	City		Reference		
	Semi-urban areas	0.202	0.850	0.663	1.091
	Village	0.457	0.927	0.759	1.132
	Migrants from countryside living in cities	**0.035**	**1.344**	**1.021**	**1.768**
Single-child	Yes		Reference		
	No	**0.003**	**0.773**	**0.651**	**0.918**
Father emotional warmth		**<0.001**	**0.973**	**0.966**	**0.98**
Father punishment		**<0.001**	**1.108**	**1.089**	**1.129**
Father interference		**<0.001**	**1.066**	**1.045**	**1.087**
Father preference		0.686	0.994	0.967	1.022
Father rejection		**<0.001**	**1.216**	**1.179**	**1.255**
Father overprotection		**<0.001**	**1.070**	**1.036**	**1.105**
Mother emotional warmth		**<0.001**	**0.975**	**0.968**	**0.981**
Mother interference		**<0.001**	**1.043**	**1.031**	**1.056**
Mother rejection		**<0.001**	**1.137**	**1.112**	**1.162**
Mother punishment		**<0.001**	**1.100**	**1.076**	**1.125**
Mother preference		0.842	1.003	0.976	1.031
Maternal health status during pregnancy	Health	Reference			
	Hypertension of pregnancy	**0.008**	**1.974**	**1.198**	**3.253**
	Other	0.976	1.021	0.263	3.959
Threatened abortion	No	Reference			
	Yes	**<0.001**	**2.284**	**1.685**	**3.094**
Mode of delivery	Natural birth	Reference			
	Cesarean delivery	**0.031**	**1.216**	**1.018**	**1.453**
	Instrumental delivery production	**<0.001**	**4.086**	**2.126**	**7.853**
Newborn health	Health	Reference			
	Neonatal asphyxia	**<0.001**	**3.635**	**2.051**	**6.442**
	Other	**0.04**	**2.726**	**1.048**	**7.093**
Birth weight	Normal birth weight	Reference			
	Low birth weight	**0.036**	**1.457**	**1.024**	**2.072**
	Macrosomia	0.889	1.046	0.555	1.972
Feeding patterns	Breast feeding	Reference			
	Artificial feeding	0.108	1.253	0.952	1.650
	Mixed feeding	0.135	1.218	0.940	1.578

### Multivariate Analysis for the Relationship Between ADHD and Variables of Maternal Pregnancy and Perinatal Period in the Total Study Population

We included all maternal events occurring during pregnancy and perinatal period in the logistic regression model 1; the results showed that TA [OR: 1.922 (1.399–2.64)] (vs. no-TA), instrumental delivery [OR: 2.238 (1.087–4.606)] (vs. natural birth) and NA [OR: 2.34 (1.234–4.436)] (vs. health) were risk factors for ADHD (Supplementary Table 1). In model 2, we further controlled for demographic variables and parental rearing style variables based on model 1; the logistic regression model shown that TA [OR: 1.707 (1.201–2.426)] (vs. no-TA) and NA [OR: 2.497 (1.225–5.09)] (vs. health) were risk factors for ADHD (Table 3).

**TABLE 3:** Results of multivariate analysis (logistic regression analysis) showing relationship between ADHD and maternal events occurring during pregnancy and perinatal period in the second model (*n* = 2638).

Variables		*P*	OR	95% CI	
Maternal health status during pregnancy	Health	Reference			
	Hypertension of pregnancy	0.463	1.273	0.668	2.424
	Other	0.679	0.715	0.145	3.512
Threatened abortion	No	Reference			
	Yes	**0.003**	**1.707**	**1.201**	**2.426**
Mode of delivery	Natural birth	Reference			
	Cesarean delivery	0.863	0.981	0.79	1.219
	Instrumental delivery	0.079	2.084	0.919	4.728
Newborn health	Health	Reference			
	Neonatal asphyxia	**0.012**	**2.497**	**1.225**	**5.09**
	Other	0.076	2.742	0.901	8.344
Birth weight	Normal birth weight	Reference			
	Low birth weight	0.426	1.19	0.775	1.827
	Macrosomia	0.548	0.809	0.404	1.618
Feeding patterns	Breastfeeding	Reference			
	Artificial feeding	0.83	1.035	0.755	1.42
	Mixed feeding	0.832	1.032	0.769	1.387

*Adjusted for demographic variables (gender of students, age of students, father’s age, father’s education level, mother’s age, mother’s education level, family economic level, place of residence, single-child) and parental rearing style (father emotional warmth, father punishment, father interference, father’s preference, father rejection, father overprotection, mother emotional warmth, mother interference, mother rejection, mother punishment and mother’s preference).

### Subgroup Analysis in Subgroups of Subjects With ADHD Disaggregated by Comorbidity (ADHD Comorbid With/Without Other Mental Disorders) and Gender

In subgroup analysis, we employed univariate analysis and multivariate analysis in the first model (Supplementary Tables 2–9). In the first model, TA [OR: 2.433 (1.682–3.518)] (vs. no-TA) and artificial feeding [OR: 1.511 (1.082–2.11)] (vs. breast feeding) were risk factors for ADHD without comorbidity (Supplementary Table 3); instrumental delivery [OR: 2.738 (1.182–6.343)] (vs. natural birth) and NA [OR: 2.781 (1.306–5.92)] (vs. health) were risk factors for ADHD with comorbidity (Supplementary Table 5); TA [OR: 2.096 (1.409–3.118)] (vs. no-TA) and NA [OR: 2.69(1.175–6.156)] (vs. health) were risk factors for male ADHD (Supplementary Table 7), while low birth weight [OR: 1.915 (1.079–3.4)] (vs. normal birth weight) was a risk factor for female ADHD (Supplementary Table 9).

Multivariate analysis in the subgroup of ADHD without/with comorbidity and gender were adjusted for demographic variables, and parental rearing style. The results of final models showed that TA [OR: 2.216 (1.458–3.369)] (vs. no-TA) was a risk factor for ADHD without comorbidity; instrumental delivery [OR: 2.748 (1.057–7.142)] (vs. natural birth) and NA [OR: 2.789 (1.222–6.361)] (vs. health) were risk factors for ADHD with comorbidity; TA (vs. no-TA) and NA (vs. health) were risk factors for ADHD among male groups [ORs: 2.232 (1.439–3.462) and 2.808 (1.115–7.068), respectively], while low birth weight (LBW) [OR: 2.054 (1.063–3.967)] (vs. normal birth weight) was a risk factor for ADHD among females groups (Table 4).

**TABLE 4:** Results of multivariate analysis (logistic regression analysis) for the subgroup of ADHD with comorbidity, disaggregated by gender in the second model.

Variables		ADHD without comorbidity				ADHD with comorbidity				Male				Female			
		P	OR	95% CI		P	OR	95% CI		P	OR	95% CI		P	OR	95% CI	
Mother pregnant situation during perinatal period	Health	Reference				Reference				Reference				Reference			
	Hypertension of pregnancy	0.382	1.412	0.651	3.059	0.505	1.341	0.565	3.183	0.178	1.751	0.774	3.96	0.479	0.615	0.16	2.359
	Other	0.832	0.805	0.107	6.022	0.878	0.846	0.099	7.216	0.68	0.648	0.083	5.087	0.937	1.117	0.071	17.651
Threatened abortion	No	Reference				Reference				Reference				Reference			
	Yes	**<0.001**	**2.216**	**1.458**	**3.369**	0.191	1.372	0.855	2.201	**<0.001**	**2.232**	**1.439**	**3.462**	0.806	0.916	0.454	1.846
Mode of delivery	Natural birth	Reference				Reference				Reference				Reference			
	Cesarean delivery	0.415	0.891	0.674	1.177	0.895	1.019	0.767	1.355	0.322	0.873	0.667	1.142	0.495	1.147	0.774	1.701
	Instrumental delivery	0.262	1.78	0.65	4.873	**0.038**	**2.748**	**1.057**	**7.142**	0.269	1.695	0.665	4.32	0.314	2.294	0.455	11.563
Newborn health	Health	Reference				Reference				Reference				Reference			
	Neonatal asphyxia	0.101	2.186	0.86	5.558	**0.015**	**2.789**	**1.222**	**6.361**	**0.028**	**2.808**	**1.115**	**7.068**	0.173	2.36	0.686	8.114
	Other	0.197	2.46	0.627	9.646	0.239	2.279	0.578	8.993	0.376	1.885	0.463	7.671	0.17	3.74	0.569	24.573
Birth weight	Normal birth weight	Reference				Reference				Reference				Reference			
	Low birth weight	0.249	1.37	0.802	2.338	0.915	1.032	0.582	1.829	0.502	0.817	0.453	1.474	**0.032**	**2.054**	**1.063**	**3.967**
	Macrosomia	0.318	0.624	0.247	1.575	0.709	0.84	0.336	2.099	0.498	0.757	0.338	1.695	0.967	0.969	0.219	4.294
Feeding patterns	Breast feeding	Reference				Reference				Reference				Reference			
	Artificial feeding	0.073	1.409	0.968	2.052	0.227	0.756	0.481	1.189	0.6	1.115	0.743	1.672	0.887	1.041	0.6	1.804
	Mixed feeding	0.891	0.973	0.659	1.437	0.627	1.095	0.759	1.581	0.824	1.043	0.718	1.515	0.604	1.146	0.684	1.92

*All models adjusted for demographic variables (gender of students, age of students, father’s age, father’s education level, mother’s age, mother’s education level, family economic level, place of residence, single-child) and parental rearing style (father emotional warmth, father punishment, father interference, father’s preference, father rejection, father overprotection, mother emotional warmth, mother interference, mother rejection, mother punishment and mother’s preference). ADHD: Attention deficit/hyperactivity disorder.*

## Discussion

The etiopathogenesis of ADHD is not clear; however, both genetic (Yuan et al., 2017) and environmental factors (pre-, peri-, and postnatal environmental factors) are believed to be involved (Mick et al., 2002; Howe, 2010; Silva et al., 2014). In some studies, the interaction between genetic and environmental factors was also found to increase the risk of ADHD (Howe, 2010). Among the environmental factors, risk factors pertaining to pregnancy and perinatal period, such as, LBW, NA, and delivery by caesarean section (Mick et al., 2002; Li et al., 2011; Curran et al., 2015; Franz et al., 2018), have attracted much attention. However, some studies have yielded conflicting results (Mimounibloch et al., 2013; Silva et al., 2014). In addition, few studies have explored the risk factors pertaining to pregnancy and perinatal period in different ADHD subgroups. In this study, we evaluated the stability of results related to the association between risk factors pertaining to pregnancy and perinatal period and ADHD in a large sample and in different ADHD subgroups. Our results indicated that TA, NA, LBW, and instrumental delivery are risk factors for ADHD.

First, we found a strong correlation between TA and ADHD in the total study population, which is not consistent with the results of previous studies (Dongol et al., 2011). This inconsistency is likely attributable to the different sample characteristics. Sliva’s study included children, adolescents, and adults; however, our study only included students in the age-group of 6–16 years (Silva et al., 2014). Additionally, we controlled for parental rearing style, which is an important postnatal environmental factor. However, Sliva’s study only included risk factors pertaining to pregnancy (Silva et al., 2014). TA is a common occurrence during pregnancy (reported incidence rate: 20–25%) (Dongol et al., 2011); it is associated with many poor pregnancy outcomes including placental abruption, preterm delivery, intrauterine growth restriction, and LBW (Basama and Crosfill, 2004; Weiss et al., 2004); all of these developments may affect brain development.

Second, in the present study, NA was found to increase the risk of ADHD in the total population, which is consistent with previous findings. For example, Linnet et al. (2005) found that Apgar score <7 at 5 min increases the risk of ADHD. Getahun et al. (2013) also found similar results on multivariate analysis. Neonatal asphyxia is a serious perinatal event that causes brain injury. Prior studies (Pagida et al., 2013; Tapia-Bustos et al., 2016) have found that hypoxia can cause long-term disturbances of dopaminergic system, and even decrease the expression of dopamine D2 receptor (Kaewsuk et al., 2009), which in turn increases the risk of ADHD. In addition, recent studies have found that asphyxia can alter *Catechol-O-methyltransferase* (*COMT*) gene expression; *COMT* gene was shown to be associated with ADHD or ADHD traits (Park et al., 2015; Akutagavamartins et al., 2016). All these findings support the notion that NA is a risk factor for ADHD.

Finally, in order to test the stability of results, we performed subgroup analysis after disaggregating the ADHD population by presence or absence of comorbidity and gender. In the subgroup analysis of ADHD population with/without comorbidity, we found that TA and NA were associated with ADHD without other comorbid mental disorders and ADHD with other comorbid mental disorders, respectively, this finding is consistent with the findings observed in the total study population. In addition, we also found that ADHD with other comorbid mental disorders was associated with more events during pregnancy and perinatal period. Instrumental delivery also increased the risk of ADHD with other comorbid mental disorders, which is a novel finding of our study. Prior studies have shown that instrumental delivery increases the risk of fetal injury, included head injuries (Doumouchtsis and Arulkumaran, 2006); however, further studies are required to explore the underlying mechanisms. On subgroup analysis disaggregated by gender, we found that TA and NA increased the risk of ADHD in male subjects, which is consistent with the results in the total ADHD population. However, only LBW was a risk factor for ADHD in female subjects. In the total ADHD population, LBW did not appear as a significant risk factor; however, it was found to be a risk factor for ADHD among females. This may be attributable to the higher proportion of males with ADHD in this study population. No study has explained the gender-based differences in the relationship between LBW and ADHD. For example, Sauver et al. (2004) and Mick et al. (2002) found no sex-based differences with respect to the association of LBW with ADHD. The association between LBW and ADHD can be explained by several reasons. First, newborns with LBW are often borne of preterm deliveries, which reduces the time for maturation of brain; this assertion is based on functional magnetic resonance imaging studies (Niwa et al., 2017). Moreover, a recent study has found that preterm birth injury affects the cholinergic system (Grothe et al., 2016), which in turn is linked to ADHD. Second, newborns with LBW are at a higher risk of morbidity, which may affect the parental rearing style and the overall growth. Third, overlapping risk gene has been found between ADHD and LBW. *MTHFR* gene is associated with infant birth weight (Wu et al., 2017) as well as with ADHD (Gokcen et al., 2011). Lastly, LBW affects gene expression, such as that of hippocampal gene (Buschdorf et al., 2016), which is a potential molecular basis for mental disorder. Some studies have found other events of ADHD related to pregnancy and the perinatal period, such as, caesarean delivery, feeding styles, and hypertension of pregnancy (Curran et al., 2015; Say et al., 2015; Pohlabeln et al., 2017). However, these findings were not confirmed in this study probably because those studies used only univariate analysis or were based on a small sample size.

### Limitations

Several limitations of this study should be acknowledged. First, despite the large sample size in this study, the cross-sectional design of our study does not permit causal inferences. Therefore, a large cohort study is required to further examine the relationship of events occurring during pregnancy and ADHD. Second, the possibility of recall bias for parental rearing attitude and behavior, and events related to pregnancy and the perinatal period cannot be ruled out. Third, parental smoking during the perinatal period has been shown to be associated with a higher risk of ADHD in the progeny (Linnet et al., 2005); however, we did not control for parental smoking during pregnancy and perinatal period in the analysis. Finally, only 3,418 questionnaires were effectively completed with respect to events related to pregnancy and the perinatal period; the lower response rate may have introduced an element of selection bias.

## Conclusion

In this study, TA, NA, instrumental delivery, and LBW were found to be associated with an increased risk of ADHD in the total study population or in subgroups of subjects with ADHD disaggregated by comorbidity and gender. Among these, TA was found to increase the risk of ADHD without other comorbid mental disorders. Instrumental delivery and NA were found to increase the risk of ADHD with other comorbid mental disorders. TA and NA were found to be risk factors for ADHD among males, but not among females. LBW was a risk factor for ADHD among females.

## Data Availability Statement

The original contributions presented in the study are included in the article/Supplementary Material, further inquiries can be directed to the corresponding author/s.

## Ethics Statement

The studies involving human participants were reviewed and approved by the Ethics Committee at the Second Xiangya Hospital. All parents or guardians and students in this study gave written informed consent.

## Author Contributions

JBL was responsible for data processing and writing of the manuscript. YQH participated in the investigation and writing of the manuscript. YMS participated in the design, investigation, and evaluation of the study. YYZ participated in data collection and analysis. TTM participated in the investigation and evaluation of the study. BX and XLC participated in the investigation and evaluation of the study. YMF participated in data processing. XRL participated in the design, investigation, and evaluation of the study and contributed to critical revision. YTX contributed to critical revision. JPL contributed to critical revision and evaluation of the study. All authors have read and approved the manuscript.

## Conflict of Interest

The authors declare that the research was conducted in the absence of any commercial or financial relationships that could be construed as a potential conflict of interest.

## Publisher’s Note

All claims expressed in this article are solely those of the authors and do not necessarily represent those of their affiliated organizations, or those of the publisher, the editors and the reviewers. Any product that may be evaluated in this article, or claim that may be made by its manufacturer, is not guaranteed or endorsed by the publisher.
