# Supplementary material for: Association of Attention Deficit/Hyperactivity Disorder With Events Occurring During Pregnancy and Perinatal Period
Source: Front Psychol. 2021 Sep 21;12:707500. doi: 10.3389/fpsyg.2021.707500 (PMC8491652; doi:10.3389/fpsyg.2021.707500)
Supplement: Supplementary file 1 [file Table_1.docx]

**Supplement Table 1. Result of logistic regression analysis showing relationship between ADHD and maternal events occurring during pregnancy and perinatal period in the first model**

| Variables | | *P* | OR | 95% CI | |
| --- | --- | --- | --- | --- | --- |
| Maternal health status during pregnancy | Health | Reference | | | |
|  | Hypertension of pregnancy | 0.465 | 1.229 | 0.707 | 2.138 |
|  | Other | 0.782 | 0.821 | 0.203 | 3.318 |
| Threatened abortion | No | Reference | | | |
|  | Yes | **<0.001** | **1.922** | **1.399** | **2.64** |
| Mode of delivery | Natural birth | Reference | | | |
|  | Cesarean delivery | 0.177 | 1.134 | 0.945 | 1.361 |
|  | Instrumental delivery | **0.029** | **2.238** | **1.087** | **4.606** |
| Newborn health | Health | Reference | | | |
|  | Neonatal asphyxia | **0.009** | **2.34** | **1.234** | **4.436** |
|  | Other | 0.099 | 2.313 | 0.854 | 6.259 |
| Birth weight | Normal birth weight | Reference | | | |
|  | Low birth weight | 0.788 | 1.055 | 0.716 | 1.553 |
|  | Macrosomia | 0.862 | 0.945 | 0.496 | 1.798 |
| Feeding patterns | Breast feeding | Reference | | | |
|  | Artificial feeding | 0.299 | 1.162 | 0.876 | 1.541 |
|  | Mixed feeding | 0.229 | 1.175 | 0.903 | 1.528 |

**Supplement Table 2. Results of univariate analysis for predicting ADHD without comorbidity**

|  | Variables | P | OR | 95% CI | |
| --- | --- | --- | --- | --- | --- |
| gender of students | Male | Reference | | | |
|  | Female | <0.001 | 0.258 | 0.2 | 0.332 |
| Age of students |  | <0.001 | 0.824 | 0.791 | 0.858 |
| Father's age | 20-29 | Reference | | | |
|  | 30-39 | 0.368 | 2.592 | 0.326 | 20.584 |
|  | 40-49 | 0.655 | 1.605 | 0.202 | 12.735 |
|  | ≥50 | 0.485 | 2.16 | 0.249 | 18.728 |
| Father's education level | Primary school educational level below | Reference | | | |
|  | Junior high school level | 0.757 | 1.052 | 0.764 | 1.448 |
|  | High school level | 0.376 | 1.169 | 0.828 | 1.65 |
|  | Junior college level | 0.980 | 1.006 | 0.643 | 1.573 |
|  | University education level | 0.401 | 0.813 | 0.501 | 1.319 |
|  | Postgraduate level | 0.799 | 0.901 | 0.403 | 2.012 |
| Mother's age | 20-29 | Reference | | | |
|  | 30-39 | 0.244 | 0.657 | 0.324 | 1.332 |
|  | 40-49 | 0.033 | 0.458 | 0.223 | 0.939 |
|  | ≥50 | 0.596 | 0.703 | 0.191 | 2.588 |
| Mother's education level | Primary school educational level below | Reference | | | |
|  | Junior high school level | 0.765 | 0.954 | 0.701 | 1.298 |
|  | High school level | 0.414 | 1.148 | 0.825 | 1.596 |
|  | Junior college level | 0.339 | 1.255 | 0.788 | 2.001 |
|  | University education level | 0.954 | 0.986 | 0.606 | 1.605 |
|  | Postgraduate level | 0.057 | 2.248 | 0.977 | 5.174 |
| Family economic level | very wealthy families | Reference | | | |
|  | relatively wealthy families | 0.077 | 0.454 | 0.189 | 1.091 |
|  | moderate families | 0.098 | 0.491 | 0.212 | 1.14 |
|  | less well-off families | 0.223 | 0.549 | 0.209 | 1.441 |
|  | very poor families | 0.378 | 2.25 | 0.37 | 13.667 |
| Place of residence | City | Reference | | | |
|  | semi-urban areas | 0.243 | 0.829 | 0.605 | 1.136 |
|  | village | 0.175 | 0.838 | 0.65 | 1.081 |
|  | migrants from countryside living in cities | 0.741 | 1.063 | 0.74 | 1.527 |
| Single-child | Yes | Reference | | | |
|  | No | <0.001 | 0.631 | 0.508 | 0.785 |
| Father emotional warmth |  | <0.001 | 0.97 | 0.961 | 0.979 |
| Father punishment |  | <0.001 | 1.101 | 1.078 | 1.125 |
| Father interference |  | <0.001 | 1.065 | 1.038 | 1.092 |
| Father preference |  | 0.949 | 1.001 | 0.967 | 1.037 |
| Father rejection |  | <0.001 | 1.201 | 1.156 | 1.247 |
| Father overprotection |  | 0.005 | 1.06 | 1.018 | 1.105 |
| Mother emotional warmth |  | <0.001 | 0.973 | 0.965 | 0.982 |
| Mother interference |  | <0.001 | 1.043 | 1.027 | 1.06 |
| Mother rejection |  | <0.001 | 1.116 | 1.086 | 1.146 |
| Mother punishment |  | <0.001 | 1.086 | 1.057 | 1.116 |
| Mother preference |  | 0.602 | 1.009 | 0.975 | 1.046 |
| Maternal health status during pregnancy | Health | Reference | | | |
|  | Hypertension of pregnancy | 0.007 | 2.261 | 1.254 | 4.077 |
|  | Other | 0.723 | 1.33 | 0.275 | 6.426 |
| Threatened abortion | No | Reference | | | |
|  | Yes | <0.001 | 2.944 | 2.076 | 4.176 |
| Mode of delivery | Natural birth | Reference | | | |
|  | Cesarean delivery | 0.034 | 1.28 | 1.019 | 1.607 |
|  | Instrumental delivery | <0.001 | 4.382 | 2.06 | 9.323 |
| Newborn health | Health | Reference | | | |
|  | Neonatal asphyxia | <0.001 | 3.772 | 1.937 | 7.346 |
|  | Other | 0.059 | 2.947 | 0.959 | 9.057 |
| Birth weight | Normal birth weight | Reference | | | |
|  | Low birth weight | 0.030 | 1.611 | 1.048 | 2.477 |
|  | Macrosomia | 0.958 | 1.022 | 0.448 | 2.334 |
| Feeding patterns | Breast feeding | Reference | | | |
|  | Artificial feeding | 0.003 | 1.637 | 1.183 | 2.264 |
|  | Mixed feeding | 0.414 | 1.153 | 0.82 | 1.621 |

**Supplement Table 3. Result of logistic regression analysis showing relationship between ADHD without comorbidity and maternal events occurring during pregnancy and perinatal period in the first model**

| Variables |  | *P* | OR | 95% CI | |
| --- | --- | --- | --- | --- | --- |
| Maternal health status during pregnancy | Health | Reference | | | |
|  | Hypertension of pregnancy | 0.467 | 1.281 | 0.658 | 2.493 |
|  | Other | 0.891 | 1.12 | 0.22 | 5.713 |
| Threatened abortion | No | Reference | | | |
|  | Yes | **<0.001** | **2.433** | **1.682** | **3.518** |
| Mode of delivery | Natural birth | Reference | | | |
|  | Cesarean delivery | 0.239 | 1.152 | 0.91 | 1.458 |
|  | Instrumental delivery | 0.089 | 2.152 | 0.89 | 5.207 |
| Newborn health | Health | Reference | | | |
|  | Neonatal asphyxia | 0.072 | 2.084 | 0.935 | 4.642 |
|  | Other | 0.188 | 2.232 | 0.676 | 7.365 |
| Birth weight | Normal birth weight | Reference | | | |
|  | Low birth weight | 0.709 | 1.096 | 0.677 | 1.775 |
|  | Macrosomia | 0.745 | 0.87 | 0.375 | 2.017 |
| Feeding patterns | Breast feeding | Reference | | | |
|  | Artificial feeding | **0.016** | **1.511** | **1.082** | **2.11** |
|  | Mixed feeding | 0.638 | 1.087 | 0.768 | 1.54 |

**Supplement Table 4. Results of univariate analysis for predicting ADHD with comorbidity**

| Variables | | *P* | OR | 95% CI | |
| --- | --- | --- | --- | --- | --- |
| Gender of students | Male | Reference | | | |
|  | Female | <0.001 | 0.477 | 0.379 | 0.601 |
| Age of students |  | <0.001 | 0.888 | 0.852 | 0.925 |
| Father's age | 20-29 | Reference | | | |
|  | 30-39 | 0.508 | 0.64 | 0.171 | 2.394 |
|  | 40-49 | 0.442 | 0.597 | 0.16 | 2.224 |
|  | ≥50 | 0.812 | 0.84 | 0.2 | 3.527 |
| Father's education level | Primary school educational level below | Reference | | | |
|  | Junior high school level | 0.073 | 3.007 | 0.904 | 10.003 |
|  | High school level | 0.054 | 3.208 | 0.983 | 10.471 |
|  | Junior college level | 0.06 | 3.133 | 0.951 | 10.319 |
|  | University education level | 0.22 | 2.172 | 0.629 | 7.503 |
|  | Postgraduate level | 0.286 | 1.975 | 0.566 | 6.895 |
| Mother's age | 20-29 | Reference | | | |
|  | 30-39 | 0.165 | 0.594 | 0.285 | 1.238 |
|  | 40-49 | 0.169 | 0.595 | 0.284 | 1.247 |
|  | ≥50 | 0.456 | 0.58 | 0.138 | 2.431 |
| Mother's education level | Primary school educational level below | Reference | | | |
|  | Junior high school level | 0.247 | 0.842 | 0.63 | 1.127 |
|  | High school level | 0.088 | 0.749 | 0.538 | 1.044 |
|  | Junior college level | 0.041 | 0.557 | 0.318 | 0.976 |
|  | University education level | 0.126 | 0.671 | 0.403 | 1.118 |
|  | Postgraduate level | 0.642 | 1.252 | 0.485 | 3.231 |
| Family economic level | very wealthy families | Reference | | | |
|  | relatively wealthy families | 0.185 | 0.519 | 0.197 | 1.367 |
|  | moderate families | 0.284 | 0.601 | 0.236 | 1.527 |
|  | less well-off families | 0.807 | 1.134 | 0.412 | 3.121 |
|  | very poor families | 0.064 | 5 | 0.91 | 27.47 |
| Place of residence | City | Reference | | | |
|  | semi-urban areas | 0.448 | 0.877 | 0.625 | 1.231 |
|  | village | 0.789 | 1.037 | 0.794 | 1.354 |
|  | migrants from countryside living in cities | 0.003 | 1.691 | 1.196 | 2.391 |
| Single-child | Yes | Reference | | | |
|  | No | 0.783 | 0.968 | 0.768 | 1.22 |
| Father emotional warmth |  | <0.001 | 0.976 | 0.967 | 0.985 |
| Father punishment |  | <0.001 | 1.104 | 1.081 | 1.129 |
| Father interference |  | <0.001 | 1.069 | 1.042 | 1.097 |
| Father preference |  | 0.486 | 0.987 | 0.952 | 1.024 |
| Father rejection |  | <0.001 | 1.217 | 1.171 | 1.264 |
| Father overprotection |  | <0.001 | 1.081 | 1.036 | 1.128 |
| Mother emotional warmth |  | <0.001 | 0.976 | 0.967 | 0.984 |
| Mother interference |  | <0.001 | 1.045 | 1.028 | 1.062 |
| Mother rejection |  | <0.001 | 1.15 | 1.119 | 1.181 |
| Mother punishment |  | <0.001 | 1.111 | 1.081 | 1.141 |
| Mother preference |  | 0.821 | 0.996 | 0.961 | 1.032 |
| Maternal health status during pregnancy | Health | Reference | | | |
|  | Hypertension of pregnancy | 0.129 | 1.674 | 0.861 | 3.255 |
|  | Other | 0.736 | 0.697 | 0.086 | 5.684 |
| Threatened abortion | No | Reference | | | |
|  | Yes | 0.024 | 1.625 | 1.066 | 2.476 |
| Mode of delivery | Natural birth |  |  | Reference | |
|  | Cesarean delivery | 0.241 | 1.152 | 0.909 | 1.458 |
|  | Instrumental delivery | 0.001 | 3.783 | 1.717 | 8.336 |
| Newborn health | Health | Reference | | | |
|  | Neonatal asphyxia | <0.001 | 3.49 | 1.747 | 6.974 |
|  | Other | 0.137 | 2.493 | 0.747 | 8.323 |
| Birth weight | Normal birth weight | Reference | | | |
|  | Low birth weight | 0.285 | 1.295 | 0.806 | 2.079 |
|  | Macrosomia | 0.87 | 1.071 | 0.469 | 2.447 |
| Feeding patterns | Breast feeding | Reference | | | |
|  | Artificial feeding | 0.493 | 0.867 | 0.577 | 1.303 |
|  | Mixed feeding | 0.137 | 1.284 | 0.924 | 1.785 |

**Supplement Table 5. Results of logistic regression analysis showing relationship of ADHD with comorbidity with maternal events occurring during pregnancy and perinatal period in the first model**

| Variables | | *P* | OR | 95% CI | |
| --- | --- | --- | --- | --- | --- |
| Maternal health status during pregnancy | Health | Reference | | | |
|  | Hypertension of pregnancy | 0.706 | 1.153 | 0.551 | 2.414 |
|  | Other | 0.757 | 0.717 | 0.087 | 5.896 |
| Threatened abortion | No | Reference | | | |
|  | Yes | 0.107 | 1.436 | 0.925 | 2.231 |
| Mode of delivery | Natural birth | Reference | | | |
|  | Cesarean delivery | 0.493 | 1.088 | 0.854 | 1.387 |
|  | Instrumental delivery | **0.019** | **2.738** | **1.182** | **6.343** |
| Newborn health | Health | Reference | | | |
|  | Neonatal asphyxia | **0.008** | **2.781** | **1.306** | **5.92** |
|  | Other | 0.311 | 1.915 | 0.545 | 6.728 |
| Birth weight | Normal birth weight | Reference | | | |
|  | Low birth weight | 0.929 | 0.977 | 0.58 | 1.645 |
|  | Macrosomia | 0.982 | 0.99 | 0.429 | 2.285 |
| Feeding patterns | Breast feeding | Reference | | | |
|  | Artificial feeding | 0.367 | 0.826 | 0.545 | 1.252 |
|  | Mixed feeding | 0.189 | 1.25 | 0.896 | 1.744 |

**Supplement Table 6. Results of univariate analysis for predicting male ADHD**

| Variables | | *P* | OR | 95% CI | |
| --- | --- | --- | --- | --- | --- |
| Age of students |  | <0.001 | 0.870 | 0.836 | 0.905 |
| Father's age | 20-29 | Reference | | | |
|  | 30-39 | 0.351 | 1.889 | 0.496 | 7.189 |
|  | 40-49 | 0.525 | 1.542 | 0.406 | 5.856 |
|  | ≥50 | 0.218 | 2.526 | 0.578 | 11.045 |
| Father's education level | Primary school educational level below | Reference | | | |
|  | Junior high school level | 0.527 | 1.108 | 0.807 | 1.52 |
|  | High school level | 0.802 | 0.957 | 0.681 | 1.346 |
|  | Junior college level | 0.499 | 0.859 | 0.552 | 1.335 |
|  | University education level | 0.006 | 0.509 | 0.313 | 0.826 |
|  | Postgraduate level | 0.106 | 0.498 | 0.214 | 1.159 |
| Mother's age | 20-29 | Reference | | | |
|  | 30-39 | 0.182 | 0.627 | 0.316 | 1.244 |
|  | 40-49 | 0.197 | 0.633 | 0.316 | 1.268 |
|  | ≥50 | 0.620 | 0.714 | 0.189 | 2.701 |
| Mother's education level | Primary school educational level below | Reference | | | |
|  | Junior high school level | 0.831 | 1.032 | 0.771 | 1.382 |
|  | High school level | 0.670 | 0.933 | 0.679 | 1.283 |
|  | Junior college level | 0.921 | 1.024 | 0.639 | 1.642 |
|  | University education level | 0.031 | 0.59 | 0.365 | 0.954 |
|  | Postgraduate level | 0.176 | 1.828 | 0.763 | 4.377 |
| Family economic level | very wealthy families | Reference | | | |
|  | relatively wealthy families | 0.105 | 0.477 | 0.195 | 1.168 |
|  | moderate families | 0.194 | 0.563 | 0.237 | 1.338 |
|  | less well-off families | 0.905 | 1.061 | 0.404 | 2.784 |
|  | very poor families | 0.109 | 6.364 | 0.662 | 61.199 |
| Place of residence | City | Reference | | | |
|  | semi-urban areas | 0.926 | 0.986 | 0.726 | 1.339 |
|  | village | 0.675 | 1.055 | 0.821 | 1.356 |
|  | migrants from countryside living in cities | 0.309 | 1.191 | 0.85 | 1.669 |
| Single-child | Yes | Reference | | | |
|  | No | 0.949 | 0.993 | 0.805 | 1.225 |
| Father emotional warmth | | <0.001 | 0.974 | 0.966 | 0.983 |
| Father punishment |  | <0.001 | 1.086 | 1.062 | 1.109 |
| Father interference |  | <0.001 | 1.058 | 1.033 | 1.083 |
| Father preference |  | 0.036 | 0.964 | 0.931 | 0.998 |
| Father rejection |  | <0.001 | 1.176 | 1.132 | 1.222 |
| Father overprotection | | 0.007 | 1.055 | 1.015 | 1.096 |
| Mother emotional warmth | | <0.001 | 0.978 | 0.97 | 0.986 |
| Mother interference |  | <0.001 | 1.039 | 1.023 | 1.054 |
| Mother rejection |  | <0.001 | 1.115 | 1.085 | 1.146 |
| Mother punishment |  | <0.001 | 1.086 | 1.056 | 1.116 |
| Mother preference |  | 0.161 | 0.976 | 0.943 | 1.01 |
| Maternal health status during pregnancy | Health | Reference | | | |
|  | Hypertension of pregnancy | 0.005 | 2.643 | 1.349 | 5.179 |
|  | Other | 0.802 | 0.804 | 0.147 | 4.406 |
| Threatened abortion | No | Reference | | | |
|  | Yes | <0.001 | 2.402 | 1.646 | 3.506 |
| Mode of delivery | Natural birth | 0.011 |  |  |  |
|  | Cesarean delivery | 0.778 | 1.032 | 0.827 | 1.288 |
|  | Instrumental delivery | 0.003 | 3.253 | 1.506 | 7.029 |
| Newborn health | Health | Reference | | | |
|  | Neonatal asphyxia | 0.001 | 3.744 | 1.768 | 7.926 |
|  | Other | 0.168 | 2.442 | 0.686 | 8.691 |
| Birth weight | Normal birth weight | Reference | | | |
|  | Low birth weight | 0.535 | 1.163 | 0.722 | 1.873 |
|  | Macrosomia | 0.610 | 0.825 | 0.395 | 1.726 |
| Feeding patterns | Breast feeding | Reference | | | |
|  | Artificial feeding | 0.027 | 1.499 | 1.048 | 2.144 |
|  | Mixed feeding | 0.318 | 1.181 | 0.852 | 1.636 |

**Supplement Table 7. Results of logistic regression analysis showing relationship between male ADHD and maternal events occurring during pregnancy and perinatal period in the first model**

| Variables | | *P* | OR | 95% CI | |
| --- | --- | --- | --- | --- | --- |
| Maternal health status during pregnancy | Health | Reference | | | |
|  | Hypertension of pregnancy | 0.082 | 1.909 | 0.921 | 3.958 |
|  | Other | 0.691 | 0.701 | 0.121 | 4.045 |
| Threatened abortion | No | Reference | | | |
|  | Yes | **<0.001** | **2.096** | **1.409** | **3.118** |
| Mode of delivery | Natural birth | Reference | | | |
|  | Cesarean delivery | 0.625 | 0.945 | 0.753 | 1.186 |
|  | Instrumental delivery | 0.24 | 1.676 | 0.708 | 3.969 |
| Newborn health | Health | Reference | | | |
|  | Neonatal asphyxia | **0.019** | **2.69** | **1.175** | **6.156** |
|  | Other | 0.364 | 1.841 | 0.493 | 6.883 |
| Birth weight | Normal birth weight | Reference | | | |
|  | Low birth weight | 0.288 | 0.747 | 0.436 | 1.279 |
|  | Macrosomia | 0.453 | 0.75 | 0.354 | 1.589 |
| Feeding patterns | Breast feeding | Reference | | | |
|  | Artificial feeding | 0.093 | 1.373 | 0.949 | 1.987 |
|  | Mixed feeding | 0.356 | 1.17 | 0.838 | 1.632 |

**Supplement Table 8. Results of univariate analysis for predicting female ADHD**

| Variables | | *P* | OR | 95% CI | |
| --- | --- | --- | --- | --- | --- |
| Age of students |  | <0.001 | 0.851 | 0.805 | 0.900 |
| Father's age | 20-29 | Reference | | | |
|  | 30-39 | 0.345 | 0.262 | 0.016 | 4.230 |
|  | 40-49 | 0.262 | 0.204 | 0.013 | 3.285 |
|  | ≥50 | 0.356 | 0.258 | 0.015 | 4.591 |
| Father's education level | Primary school educational level below | Reference | | | |
|  | Junior high school level | 0.504 | 0.867 | 0.571 | 1.317 |
|  | High school level | 0.561 | 1.145 | 0.725 | 1.81 |
|  | Junior college level | 0.246 | 0.676 | 0.349 | 1.309 |
|  | University education level | 0.642 | 1.157 | 0.626 | 2.137 |
|  | Postgraduate level | 0.582 | 0.7 | 0.197 | 2.493 |
| Mother's age | 20-29 | Reference | | | |
|  | 30-39 | 0.660 | 0.773 | 0.245 | 2.437 |
|  | 40-49 | 0.327 | 0.561 | 0.177 | 1.783 |
|  | ≥50 | 0.769 | 0.75 | 0.11 | 5.109 |
| Mother's education level | Primary school educational level below | Reference | | | |
|  | Junior high school level | 0.085 | 0.704 | 0.472 | 1.049 |
|  | High school level | 0.504 | 0.86 | 0.553 | 1.338 |
|  | Junior college level | 0.147 | 0.578 | 0.275 | 1.212 |
|  | University education level | 0.400 | 1.31 | 0.699 | 2.454 |
|  | Postgraduate level | 0.779 | 1.213 | 0.316 | 4.659 |
| Family economic level | very wealthy families | Reference | | | |
|  | relatively wealthy families | 0.423 | 0.569 | 0.143 | 2.263 |
|  | moderate families | 0.467 | 0.609 | 0.16 | 2.319 |
|  | less well-off families | 0.397 | 0.522 | 0.116 | 2.355 |
|  | very poor families | 0.837 | 1.333 | 0.086 | 20.707 |
| Place of residence | City | Reference | | | |
|  | semi-urban areas | 0.099 | 0.672 | 0.418 | 1.078 |
|  | village | 0.557 | 0.9 | 0.632 | 1.281 |
|  | migrants from countryside living in cities | 0.053 | 1.624 | 0.993 | 2.656 |
| Single-child | Yes | Reference | | | |
|  | No | 0.212 | 0.81 | 0.581 | 1.128 |
| Father emotional warmth |  | <0.001 | 0.968 | 0.955 | 0.98 |
| Father punishment |  | <0.001 | 1.118 | 1.081 | 1.157 |
| Father interference |  | 0.058 | 1.038 | 0.999 | 1.08 |
| Father preference |  | 0.181 | 1.034 | 0.985 | 1.086 |
| Father rejection |  | <0.001 | 1.226 | 1.159 | 1.296 |
| Father overprotection |  | 0.126 | 1.049 | 0.987 | 1.116 |
| Mother emotional warmth |  | <0.001 | 0.968 | 0.956 | 0.979 |
| Mother interference |  | 0.002 | 1.036 | 1.013 | 1.059 |
| Mother rejection |  | <0.001 | 1.149 | 1.106 | 1.194 |
| Mother punishment |  | <0.001 | 1.099 | 1.056 | 1.143 |
| Mother preference |  | 0.149 | 1.037 | 0.987 | 1.088 |
| Maternal health status during pregnancy | Health | Reference | | | |
|  | Hypertension of pregnancy | 0.607 | 1.273 | 0.508 | 3.194 |
|  | Other | 0.732 | 1.485 | 0.154 | 14.352 |
| Threatened abortion | No | Reference | | | |
|  | Yes | 0.110 | 1.619 | 0.897 | 2.923 |
| Mode of delivery | Natural birth | Reference | | | |
|  | Cesarean delivery | 0.031 | 1.42 | 1.033 | 1.953 |
|  | Instrumental delivery | 0.041 | 4 | 1.06 | 15.088 |
| Newborn health | Health | Reference | | | |
|  | Neonatal asphyxia | 0.020 | 3.2 | 1.204 | 8.507 |
|  | Other | 0.108 | 3.429 | 0.761 | 15.437 |
| Birth weight | Normal birth weight | Reference | | | |
|  | Low birth weight | 0.002 | 2.304 | 1.351 | 3.929 |
|  | Macrosomia | 0.702 | 1.285 | 0.355 | 4.652 |
| Feeding patterns | Breast feeding | Reference | | | |
|  | Artificial feeding | 0.793 | 1.067 | 0.657 | 1.732 |
|  | Mixed feeding | 0.257 | 1.299 | 0.826 | 2.042 |

**Supplement Table 9. Results of logistic regression analysis showing relationship between female ADHD and maternal events occurring during pregnancy and perinatal period in the first model**

| Variables | | *P* | OR | 95% CI | |
| --- | --- | --- | --- | --- | --- |
| Maternal health status during pregnancy | Health | Reference | | | |
|  | Hypertension of pregnancy | 0.368 | 0.61 | 0.208 | 1.79 |
|  | Other | 0.911 | 1.147 | 0.104 | 12.649 |
| Threatened abortion | No | Reference | | | |
|  | Yes | 0.374 | 1.325 | 0.712 | 2.468 |
| Mode of delivery | Natural birth | Reference | | | |
|  | Cesarean delivery | 0.053 | 1.384 | 0.996 | 1.922 |
|  | Instrumental delivery | 0.21 | 2.484 | 0.598 | 10.32 |
| Newborn health | Health | Reference | | | |
|  | Neonatal asphyxia | 0.223 | 2.015 | 0.653 | 6.219 |
|  | Other | 0.118 | 3.563 | 0.725 | 17.505 |
| Birth weight | Normal birth weight | Reference | | | |
|  | Low birth weight | **0.027** | **1.915** | **1.079** | **3.4** |
|  | Macrosomia | 0.813 | 1.17 | 0.319 | 4.288 |
| Feeding patterns | Breast feeding | Reference | | | |
|  | Artificial feeding | 0.944 | 1.018 | 0.62 | 1.671 |
|  | Mixed feeding | 0.362 | 1.239 | 0.782 | 1.961 |
